# Homocysteine and psoriasis

**DOI:** 10.1042/BSR20190867

**Published:** 2019-11-22

**Authors:** Xiran Lin, Xianmin Meng, Zhiqi Song

**Affiliations:** 1 Department of Dermatology, the First Affiliated Hospital of Dalian Medical University. 222 Zhongshan Lu, Dalian 116011, China; 2 Department of Pathology and laboratory medicine, Axia Women’s Health. 450.Cresson BLVD. Oaks, PA 19456, U.S.A.

**Keywords:** folic acid, homocysteine, hydrogen sulfide, psoriasis, vitamin B12

## Abstract

Psoriasis is caused by a complex interplay among the immune system, genetic background, autoantigens, and environmental factors. Recent studies have demonstrated that patients with psoriasis have a significantly higher serum homocysteine (Hcy) level and a higher prevalence of hyperhomocysteinaemia (HHcy). Insufficiency of folic acid and vitamin B_12_ can be a cause of HHcy in psoriasis. Hcy may promote the immuno-inflammatory process in the pathogenesis of psoriasis by activating Th1 and Th17 cells and neutrophils, while suppressing regulatory T cells. Moreover, Hcy can drive the immuno-inflammatory process by enhancing the production of the pro-inflammatory cytokines in related to psoriasis. Hcy can induce nuclear factor kappa B activation, which is critical in the immunopathogenesis of psoriasis. There may be a link between the oxidative stress state in psoriasis and the effect of HHcy. Hydrogen sulfide (H_2_S) may play a protective role in the pathogenesis of psoriasis and the deficiency of H_2_S in psoriasis may be caused by HHcy. As the role of Hcy in the pathogenesis of psoriasis is most likely established, Hcy can be a potential therapeutic target for the treatment of psoriasis. Systemic folinate calcium, a folic acid derivative, and topical vitamin B12 have found to be effective in treating psoriasis.

## Introduction

Psoriasis is a chronic inflammatory disease with a worldwide prevalence of 2–3% [1]. Psoriasis is caused by a complex interplay among the immune system, genetic background, autoantigens, and environmental factors [2]. Notably, psoriasis is associated with increased risk for cardiovascular comorbidities [3], and hyperhomocysteinaemia (HHcy) has been recognized as an independent risk factor for the presence of cardiovascular diseases [4]. Therefore, the relation between psoriasis and homocysteine (Hcy) has attracted attention.

## Hcy and Hcy metabolism

Hcy is a sulphur-containing amino acid. The internationally accepted biological reference interval (normal range) of plasma Hcy is 5–15 micromol/l [5]. Of the total plasma Hcy, 80–90% exists in a protein-bound form, approximately 10–20% is in an oxidized form, and only less than 1% exists as a free, reduced amino acid [6,7].

**Figure 1:**
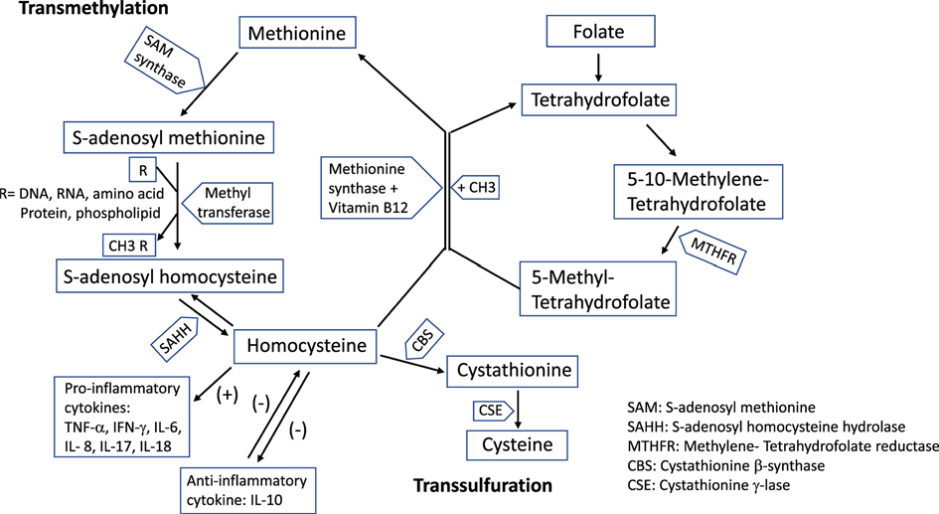
Homocysteine metabolism, role of folic acid and vitamin B_12_, and interaction with cytokines Transmethylation of methionine: S-adenosyl-L-methionine (SAM) synthase catalyzes the reaction of methionine to form SAM; SAM is converted into S-adenosyl-L-homocysteine (SAH) via methyltransferase-catalyzed methyl transfer reaction, donating the methyl group to acceptor molecules (DNA, RNA, amino acids, proteins, phospholipids etc.); SAH is metabolized by SAH hydrolase to from Hcy. Hcy can be resynthesized into SAH by SAH hydrolase; re‐methylated to form methionine (folate in the form of 5‐methyl tetrahydrofolate, derived from 5,10‐methylene tetrahydrofolate reductase catalyzed tetrahydrofolate modification, donating a methyl group to homocysteine catalyzed by vitamin B12‐dependent methionine synthase); or metabolized to form cysteine via trans-sulphuration, sequentially catalyzed by cystathionine β-synthase (CBS) and cystathionine γ-lyase (CSE). Homocysteine up-regulates pro-inflammatory cytokines and negatively interacts with anti-inflammatory cytokine IL-10.

Hcy is produced in all human tissues through the transmethylation of methionine with three steps. S-adenosyl-L-methionine (SAM) synthase catalyzes the reaction of methionine with ATP to form SAM. SAM is converted into S-adenosyl-L-homocysteine (SAH) via a methyltransferase-catalyzed methyl transfer reaction, donating the methyl group to acceptor molecules (DNA, RNA, amino acids, proteins, phospholipids etc.). Finally, SAH is rapidly metabolized by SAH hydrolase to adenosine and Hcy. The disposal of Hcy involves many pathways. In first pathway, approximately 50% of Hcy is re‐methylated to form methionine via two distinct mechanisms: folate/vitamin B12‐dependent re-methylation and folate/vitamin B12‐independent re‐methylation. In the folate/vitamin B12‐dependent mechanism, folate in the form of 5‐methyl tetrahydrofolate, derived from 5,10‐methylene tetrahydrofolate reductase catalyzed tetrahydrofolate modification, donates a methyl group to Hcy catalyzed by the vitamin B12‐dependent enzyme methionine synthase to form methionine. In second pathway, Hcy is resynthesized into SAH through the reversal of SAH hydrolase activity. In third pathway, Hcy is metabolized to form cysteine via trans-sulphuration, sequentially catalyzed by vitamin B6-dependent enzymes cystathionine β-synthase (CBS) and cystathionine γ-lyase (CSE). HHcy is the result of increased production and/or decreased disposal of Hcy [6,7] (Figure 1).

## Hcy levels in patients with psoriasis

The majority of the studies in the literature demonstrated that patients with psoriasis have plasma/serum Hcy levels significantly higher than controls [8–18]. Among them, some reported that the severity of psoriasis assessed according to psoriasis area and severity index (PASI) directly correlate with plasma/serum Hcy levels [10,15,17,18].

Only a minority of studies reported that Hcy levels do not differ significantly between patients with psoriasis and healthy controls. [19–22]

Very recently, a systematic review and meta-analysis demonstrated that, compared with controls, patients with psoriasis had a significantly higher serum Hcy level and a higher prevalence of HHcy [23]. Therefore, a link between HHcy and psoriasis is established.

## Possible causes of high Hcy levels in psoriasis

Generally speaking, deficiencies or genetic polymorphisms in the enzymes taking part in Hcy metabolism and insufficient amounts of cofactors are a major cause of HHcy, and excessive methionine intake, certain diseases, and certain drugs represent additional causes [24]. In psoriasis, lack of folic acid and vitamin B12 can be a cause of HHcy, while smoking, alcohol consumption, and obesity are associated factors.

### Folic acid and vitamin B12

In human body, approximately 50% of Hcy is re-methylated to form methionine, and this remethylation reaction requires a folate coenzyme. Thus, folic acid deficiency can result in a buildup of Hcy. Studies have demonstrated that plasma levels of folic acid are lower in psoriatic patients than in controls [9–12], and plasma/serum Hcy levels inversely correlate with serum folic acid levels in psoriatic patients [10,19]. The increase of Hcy can be caused by the decrease of folic acid.

One proposed mechanism of decreased folate in psoriasis may associated with inflammatory changes in intestinal mucosa, which causes reduced absorption of dietary folate [10]. Another rational explanation may be related to rapid skin turn over in psoriasis with subsequent increased consumption of folate leading to low serum folate [13,25,26].

In the conversion of Hcy into methionine, folate in the form of N-5-methyl tetrahydrofolate donates a methyl group to Hcy in the remethylation catalyzed by methionine synthase in concert with vitamin B_12_ [9]. Lower levels of vitamin B_12_ were found in patients with HHcy compared with patients with a normal value of Hcy [12]. This suggests that deficiency of vitamin B12 may also contribute to the increase of Hcy in psoriasis.

### Smoking and alcohol consumption

Studies have revealed a significant increase in Hcy levels in smokers compared with non-smokers [27–30]. Meanwhile, a systematic literature review and meta-analysis identified significant association between the prevalence of smoking and psoriasis [31].

A number of studies have showed that consumption of alcohol correlates with elevated plasma/serum Hcy [32–40]. According to a systematic literature review, excessive drinking was more prevalent among psoriasis patients than in the general population and psoriasis patients consumed more alcohol than the controls [41]. Therefore, smoking and alcohol consumption can be proposed as common factors between HHcy and psoriasis.

### Obesity

The association between psoriasis and obesity has been established [42,43]. A number of studies have demonstrated that Hcy levels are significantly increased in obese individuals when compared with the normal weight population [44–49]. Thus, obesity may also be recognized as one of the associated factors between elevated Hcy levels and psoriasis.

## Possible roles of Hcy in the initiation, maintenance, exacerbation, and remission of psoriasis

It is well known that dendritic cells (DCs) play a major role in the initial stages of psoriasis. The activation of plasmacytoid DC is the key in starting the development of the psoriatic lesions, leading to the recruitment and activation of myeloid DCs and T cells that are responsible for lesion maintenance [50,51]. Reactive oxygen species (ROS) may play an important role in activation of DCs [52] and increase the DC ability to activate CD4^+^ T cells [53]. Oxidative stress (OS) has been suggested as a primary mechanism responsible for HHcy-related pathogenesis [54]. Therefore in psoriasis, increased Hcy may facilitate the disease initiation and maintenance via increasing DC activation by ROS.

Many events and factors are associated with the onset and worsening of psoriasis. Among these, some are related to Hcy. For example, it was shown that mental stress elevates the plasma total Hcy level in young men [55]. Smoking increases the risk of psoriasis and its severity. Alcohol use and abuse are also associated with psoriasis [56]. As we mentioned above, smoking and alcohol consumption can be proposed as common factors between HHcy and psoriasis. Some factors contributing to remission of psoriasis are negatively related to Hcy. For example, a majority of psoriatic patients experience improvement during pregnancy [57]. In normal pregnancy, Hcy concentrations fall [58]. Psoriasis severity is negatively correlated with adherence to the Mediterranean diet [59], and adherence to the Mediterranean diet is inversely correlated with Hcy levels [60].

## Possible roles of Hcy in the immuno-pathogenesis of psoriasis

Although HHcy has been associated with psoriasis, the role of Hcy in the pathogenesis of psoriasis remains to be elucidated. As psoriasis is considered as an immune-mediated disease, we first review the literature on Hcy’s role in various immuno-inflammatory factors relating to the pathogenesis of psoriasis.

### Th1 and Th17 cells

Research has unequivocally shown that psoriasis represents a bona fide T-cell mediated disease [2], and Hcy indeed increases the proliferation of lectin stimulated T cells [61]. Psoriasis was originally considered a Th1-mediated skin disease, whereas, in recent years, the focus has been shifted to Th17 cells and to other IL-17-producing cell types [62]. Th1 and Th17 cells may collaboratively interact with each other and contribute to the autoimmune disease pathogenesis [63]. Several lines of evidence have suggested that Hcy may exert a stimulatory effect on T cell functions and induce cytokine secretion, especially Th1-type cytokines, including IFN-γ and IL-2 [64–67]. In lamina propria lymphocytes in colonic mucosa of Wistar rats, Hcy promoted the differentiation of CD4^+^ T cells into Th17 cells in a dose-dependent manner [68]. *In vitro*, Hcy treatment resulted in enhanced IL-17 secretion by T cells [66]. Moreover, *ex vivo* ELISA assay revealed significantly increased IL-2, IFN-γ [66,67], and IL-17 [66] levels in activated splenic T cells from apoE-/- mice with HHcy compared with mice without HHcy. Taken together, above-mentioned studies have demonstrated that Hcy is a activator of Th1 and Th17 cells. HHcy may contribute to the overactivation of Th1 and Th17 cells in the pathogenesis of psoriasis.

### Regulatory T cells (Tregs)

The activity of Th1 and Th17 cells is modulated by Tregs, which are able to inhibit the immunological response and to maintain the cutaneous immunological homeostasis, thus preventing autoimmunity against self-antigens. Several studies demonstrate that the function of Tregs is impaired in psoriasis and treatments for psoriasis may increase the number and activity of Treg [69]. Studies showed that HHcy impaired the suppressive function of Tregs *in vitro* and *in vivo*. Feng et al. [66] investigated the role of Tregs in HHcy-accelerated atherosclerosis in apoE-/- mice. Cotransfer of normal Tregs significantly attenuated atherosclerotic lesion size and infiltration of T cells and macrophages into plaque. Furthermore, Treg cotransfer reversed HHcy-accelerated proliferation of T cells. With a clinically relevant level of plasma Hcy (30 μM), the proportion of Tregs and suppressive activity in splenic T cells were reduced [66]. This was associated with reduced mRNA and protein expression of Foxp3, a factor governing Treg development and function. In addition, Hcy significantly attenuated the proportion and suppressive effects of Tregs *in vitro*. The author concluded that HHcy suppresses the function of Tregs, which may be responsible for HHcy-accelerated atherosclerosis in apoE-/- mice. Of note, psoriasis shares striking similarities with atherosclerosis, such as the histological features and a immunoinflammatory cascade involving Th1, Th17, Tregs, and downstream expression of cytokines [70]. Therefore, HHcy could also suppress the function of Tregs in psoriasis.

### Neutrophil

In early phase of psoriasis, neutrophils infiltrate the dermis and subsequently migrate into epidermis to form Munro’s microabscesses, a histopathological feature of the disease [71]. Alvarez-Maqueda et al. [72] investigated the role of Hcy in essential functions of human neutrophils and showed that Hcy increases superoxide anion release by neutrophils to the extracellular medium and increases intracellular H_2_O_2_ production by neutrophils. Hcy enhances the activation and phosphorylation of mitogen-activated protein kinases. The migration of neutrophils is increased by Hcy [72]. Thus, HHcy might contribute to the formation of psoriatic lesions by enhancing the activity and migration of neutrophils.

### Cytokines

In psoriasis, a number of pro-inflammatory cytokines including TNF-α, IFN-γ, IL-6, IL-8, IL-12, IL-17, and IL-18 are overexpressed, driving the pathogenesis in the disease. Three cytokines, namely TNF-α, IL-17 and IFN-γ, play the key role in the development of psoriatic lesions [73]. Studies have shown that the production of these pro-inflammatory cytokines may be affected by Hcy (Figure 1).

In a cross-sectional study on healthy men, plasma total Hcy concentration showed positive correlation with IL-1β, TNF-α, and IL-6 [74]. In experimental rats, moderate exogenous HHcy was associated with increase in TNF-α, IFN-γ, and IL-17α concentrations in the serum and in mononuclear cells [75]. Acute administration of Hcy and chronic HHcy significantly increased proinflammatory cytokines TNF-α, IL-1β, and IL-6 in serum of rats [76,77]. Mild HHcy induced in rats increased IL-6 levels in serum [78]. In a mouse model, HHcy elevated plasma levels of TNF-α and IL-6 [79]. Aso et al. [80] found that plasma total Hcy levels were significantly higher in Type 2 diabetic patients with high plasma IL-18 concentration than in those with normal plasma IL-18 concentration. McLachlan et al. [81] reported that Hcy is positively associated with plasma IL-18 concentrations in coronary artery bypass surgery patients. Tso et al. [82] found that plasma concentrations of IL-18 correlated positively and significantly with Hcy in SLE patients. Wang et al. [83] detected increased plasma levels of IL-1β and IL-18 in HHcy mice as compared with control mice. *In vitro* studies showed that Hcy can induce IL-1β [84], TNF-α, IL-6, IL-12 [85], and IL-8 [86,85] production by human peripheral blood monocytes.

The fact of Hcy enhancing the production of pro-inflammatory cytokines which indeed overexpress in psoriasis suggests the role of Hcy in psoriasis pathogenesis.

Treg cells interact with other cells by producing anti-inflammatory cytokines including IL-10, IL-35, and TGF-β [87]. Deficiency of anti-inflammatory cytokines IL-10 [88] and IL-35 [89] in patients with psoriasis are essential factor in pathogenesis. IL-10 has an anti-inflammatory effect, inhibiting the production of pro-inflammatory cytokines [88]. Matrix metalloproteinases (MMPs) are thought to be associated with the pathogenesis and spread of psoriatic disease [90]. Plasma levels of MMP-9 was significantly elevated in psoriasis patients compared with healthy individuals [90]. Hyperhomocysteinemic subjects also had raised serum levels of MMP-9 comparing healthy controls, and although IL-10 markedly suppressed MMP-9 release from PBMCs in controls, no or only minor effect was seen in hyperhomocysteinemic subjects [91]. These findings suggest that Hcy can play a role in psoriasis via attenuating the inhibitory effect of IL-10 on MMP-9 production. Studies in mice showed that administration of IL-10 reduced serum Hcy levels [92], suggesting a negative impact of IL-10 on Hcy (Figure 1). TGF-β is an important regulator in maintaining immune homeostasis. However, the role of TGF-β in psoriasis is still not fully explained [93].

### Nuclear factor κB (NF-κB)

NF-κB is a transcription factor that orchestrates inflammation and other complex biological processes. It is a key regulatory element in a variety of immune and inflammatory pathways, in cellular proliferation and differentiation and in apoptosis. NF-κB is a crucial mediator involved in the pathogenesis of psoriasis which is marked by elevated levels of active, phosphorylated NF-κB [94].

Studies have observed that Hcy can induce NF-κB activation. In human aorta vascular smooth-muscle cells, Hcy significantly activated NF-κB [95]. In human monocytic cell (THP-1)-derived macrophages, Hcy at pathological concentration stimulated NF-κB activation [96]. In the endothelium of aortas isolated from HHcy rats, activated form of NF-κB was detected [97]. In a model of heart failure established by high methionine diet treatment, plasmatic Hcy level was elevated and an association between HHcy and activation of NF-κB was disclosed [98].

Activation of NF-κB may play a key role in epidermal hyperproliferation in psoriasis [99]. Moreover, NF-κB is a central mediator of pro-inflammatory gene induction and functions in both innate and adaptive immune cells [100]. Therefore, the effect of Hcy on NF-κB activation may contribute to the immunopathogenesis of psoriasis.

## Hcy and OS in psoriasis

OS is defined as an imbalance between the production of reactive species and antioxidant defences. It can result from increased production of ROS and reduced levels of antioxidants. OS has been suggested as a primary mechanism responsible for HHcy related pathogenesis. ROS are generated during oxidation of the free thiol group of Hcy. Hcy can inhibit the activity of cellular antioxidant enzymes, disrupt extracellular superoxide dismutase, and activate nicotinamide adenine dinucleotide phosphate oxidase [101].

Published studies have provided plenty of evidences that psoriasis is in a state of OS, which may play a critical role in the pathogenesis of the disease [102]. There may be a link between the OS state in psoriasis and the effect of HHcy.

## Hcy and hydrogen sulfide (H_2_S) in psoriasis

H_2_S is a colorless gas with a strong odor that until recently was only considered to be a toxic environmental pollutant with little or no physiological significance. However, in recent years its roles as a major player in many mammalian biological systems have been demonstrated [103]. Under physiological conditions, Hcy metabolizes to produce cysteine, which is a substrate of CBS and CSE for endogenous production of H_2_S. Also, endogenous H_2_S is generated from Hcy metabolism through trans-sulfuration pathway, catalyzed by CSE and CBS. Hcy and cysteine both are substrates for H_2_S generation. In pathological condition, HHcy inhibits CSE enzyme activity and reduces endogenous production of H_2_S. Besides, Hcy can compete for binding to CSE with cysteine, thereby decreasing H_2_S production from cysteine through substrate inhibition. Therefore, it could be supposed that during HHcy, H_2_S production will be diminished [104]. Indeed, in HHcy animal models, decreased H_2_S levels [105–107], and decreased CSE activity [105,106] were concomitantly observed. In addition to the generation of H_2_S, CSE and CBS can also be considered as Hcy-clearing enzymes. Depletion of both CBS and CSE causes HHcy [108]. H_2_S donor may also have a Hcy lowering effect. For example, a H_2_S donor—sodium hydrosulfide (NaHS) significantly reduced concentration of Hcy in rats with HHcy [109]. The Hcy lowering effect of H_2_S donor could result from the stimulation of the trans-sulphuration pathway by activating CSE and CBS. For example, administration of NaHS significantly up-regulates the gene expression of CSE and GSH in C_2_C_12_ mouse myotubes. Additionally, it reduces Hcy [110].

Alshorafa et al. [111] investigated the relationship of H_2_S with psoriasis and showed that serum H_2_S levels in psoriasis patients were significantly lower than those of healthy controls and negatively correlated with clinical disease severity. Exogenous H_2_S inhibited the TNF-α-mediated up-regulation of NO, IL-6, and IL-8 in a dose-dependent manner. In addition, H_2_S inhibited TNF-α-mediated activation of p38, extracellular-signal-regulated kinase and NF-κB. The authors concluded that H_2_S may play a protective role in the pathogenesis of psoriasis and H_2_S-releasing agents may be promising therapeutics for psoriasis. The deficiency of H_2_S in psoriasis may be caused by HHcy.

## Relationship of psoriasis and psoriatic arthritis (PsA)

PsA is a major comorbidity of psoriasis. the reported proportion of PsA among psoriasis patients ranges from 7% to 26%. Available literature suggests that the highest yield clinical features indicating increased risk for PsA include the following: increased psoriasis severity; positive family history for psoriasis or PsA; patient history of musculoskeletal pain, morning stiffness, fatigue, and difficulty with activities of daily living; presence of scalp, intergluteal, or perianal psoriasis; nail dystrophy; and dactylitis [112]. The frequency of HLA-B27 is reportedly higher among patients with PsA. HLA B27 has been specifically associated with PsA in case–control studies that compared PsA patients with psoriasis patients. HLA-B27 has been shown to be a stronger genetic marker for PsA than for psoriasis. HLA-B27 is an independent risk allele for PsA that is unrelated to skin disease. Recent population case–control studies with adequate patient groups and replication cohorts, as well as confirmation studies in family pedigrees through the use of modern molecular typing methods, have reinforced the aetiological role of this allele in PsA [113]. High levels of Hcy have been documented in a small number of patients with PsA [114].

## A potential therapeutic regimen for psoriasis and possible role of methotrexate (MTX) and cyclosporine

Mild-to-moderate psoriasis can be treated topically with a combination of glucocorticoids, vitamin D analogues, and phototherapy. Moderate-to-severe psoriasis often requires systemic treatment. MTX, cyclosporin A, and retinoids are traditional systemic treatment options for psoriasis. Dimethyl fumarate and apremilast are newer drugs that have been approved for psoriasis. Biologics are different from the above-described systemic therapies in that they target specific inflammatory pathways. Biologics presently target two pathways crucial in the development and chronicity of the psoriatic plaque: the IL-23/Th17 axis and TNF-α-signaling. There are currently four drugs in TNF-α inhibitors: etanercept, infliximab, adalimumab, and certolizumab. Ustekinumab is a monoclonal antibody directed against the p40 subunit of IL-23. So far, three human monoclonal antibodies targeting IL-17 are available. Secukinumab and ixekizumab block IL-17A; whereas brodalumab is directed against the IL-17 receptor A [115].

The possible role of MTX in psoriasis is related to its role in inhibiting dihydrofolate reductase (DHFR) and, therefore, in the activation of folic acid. This leads to the inhibition of the synthesis of DNA. By inhibiting DNA synthesis, MTX limits epithelial hyperplasia, reinforces the apoptosis of activated T cells, and inhibits the chemotaxis of neutrophils. In addition, the drug decreases the synthesis of a range of proinflammatory cytokines such as TNF-α and IL-1. Folic acid supplementation may prevent or reduce a range of adverse reactions associated with MTX treatment of psoriasis [116]. In patients with rheumatoid arthritis (RA), during MTX treatment a significant rise in plasma Hcy was seen. In general, this effect could be reversed by folate administration [117]. Presumably, MTX may also increase plasma Hcy in psoriasis patients. It is well established that folic acid supplementation has a role in the treatment of psoriasis in conjunction with MTX treatment [118]. Plasma vitamine B_12_ levels were not affected by MTX treatment in RA patients [119].

The possible role of cyclosporine is related to its selective action on T cells. Cyclosporine inhibits the activity of calcineurin phosphatase, resulting in failing transportation of nuclear factor of activated T cells to the nucleus for transcription of genes encoding IL-2, which is necessary for full activation of the T-cell pathway. Consequently, cyclosporine depletes lymphocytes and macrophages in the epidermis and dermis and inhibits the activation of T cells. Cyclosporine also inhibits keratinocyte hyperproliferation [120]. Elevated serum Hcy levels were reported in cyclosporine‐treated renal transplant recipients. Cyclosporine may interfere with folate-assisted remethylation of Hcy. The HHcy of cyclosporine-treated patients responded to treatment with folic acid [121]. Liver transplant recipients treated with cyclosporine had higher plasma Hcy concentrations. Cyclosporine might interfere with the Hcy clearance [122].

## Targeting Hcy as a strategy for treatment of psoriasis

### Physical exercise

As obesity may also be one of the factors contributing to elevated Hcy levels in psoriasis and physical exercise can induce changes in protein and amino acid metabolism, the impact of daily physical activity and training programs on the status of Hcy in the body is worth noting. In a systematic review including 34 studies, correlative and comparative studies of Hcy levels revealed lower levels in patients engaged in greater quantities of daily physical activity. Regarding the acute effects of exercise, all studies reported increased Hcy levels. Concerning intervention studies with training programs, aerobic training programs used different methods and analyses that complicate making any conclusion, though resistance training programs induced decreased Hcy levels. In conclusion, this review suggests that greater daily physical activity is associated with lower Hcy levels and that exercise programs could positively affect Hcy control [123].

### Folic acid

In the literature, supplementation with folic acid to lower Hcy levels has been studied. However, there is a scarcity of literature describing the effect of folate on psoriasis.

Dawson et al. [124] carried out a double blind trial of folic acid therapy (took one 5 mg tablet of folic acid twice daily) on psoriasis in 21 patients over a period of 6 weeks. The results indicate neither a beneficial nor an adverse effect. Aronson [125] reported that a patient with plaque psoriasis on 10 mg lisinopril did not improve on vitamins B12 and B6 alone. When folic acid (FA) 5 mg daily was added PASI improved by 50%. Three cases of plaque psoriasis flared when given 1–2 mg (FA), 100 mg vitamin B6 and 1000 mcg daily B12. When daily FA was increased to 4–7 mg daily, all three cases were improved.

Gisondi et al. [126] proposed that folic acid appears as a reasonable therapeutic option in patients with moderate-to-severe psoriasis who have concomitant HHcy, low plasma folate and additional cardiovascular risk factors. Folinate calcium is a folic acid derivative, a reduced form of folic acid which can be rapidly absorbed after administered orally [127]. Carlesimo et al. [128] treated 30 patients affected by active chronic plaque psoriasis associated with other disorders: hypertension, diabetes, dyslipidemia and obesity, with oral folinate calcium 15 mg once daily for a variable period based on the patient’s clinical response. After the therapy a significant reduction of PASI mean values was observed (from 22.78 to 7.92), indicating an improvement of the clinical condition. Simultaneously, a reduction of plasma homocysteine levels and an increase in plasma folic acid levels were observed. The authors concluded that preliminary results support the effectiveness and tolerability of folinate calcium treatment in psoriasis and suggested to continue this study.

### Vitamin B12

Deficiency of vitamin B12 may also contribute to the increase of Hcy in psoriasis. Back to 1950s, three published observational reports of systemic vitamin B12 treatment for psoriasis gave inconsistent results [129]. A double-blind controlled trial of unselected cases published in 1962 gave no support to the belief that systemic vitamin B12 is of any value in the treatment of psoriasis, single or combined with a bland ointment, a dithranol regime, or a modified Goeckermann regime [129]. However, it was reported that injection of vitamin B12 into psoriatic lesions showed regression of the lesion in 6 of 8 cases, compared with control group of normal saline. The vitamin B12 was injected into one lesion only in each patient, and no change was noted in other lesions. Where regression took place, a smooth, slightly pink skin resulted [130]. The fact that vitamin B12 level is lower in psoriatic than non-psoriatic skin and active lesions have lower levels than healed lesions [131] may explain the effect of intralesional vitamin B12 on psoriasis.

Since up to 90% of one dose of systemic vitamin B12 is eliminated by the renal pathway within 48 h and is therefore not available at the skin lesions, Stücker et al. [132] considered cutaneous application as the most appropriate way of administration and first carried out a randomized, prospective clinical trial to evaluated the effects of a vitamin B12 cream containing avocado oil in 13 patients against the vitamin D3 analog calcipotriol in an intraindividual right/left-side comparison. After 12 weeks, the PASI score showed no significant differences between the two treatments. While the efficacy of the calcipotriol preparation reached a maximum in the first 4 weeks and then began to subside, the effects of the vitamin B12 cream remained at a constant level over the whole observation period. Moreover, the investigator and patients assessed the tolerability of the vitamin B12 cream as significantly better in comparison with that of calcipotriol. Very recently, Del Duca et al. [133] reported a randomized, controlled, single-blind, intra-patient left- to right-side trial comparing the efficacy and safety of vitamin B_12_-containing ointment (0.07% cyanocobalamin in a w/o formulation with 20% avocado oil) (M-treatment) with a glycerol-petrolatum-based emollient cream (C-treatment) in 24 patients with mild-to-moderate plaque psoriasis for a period over 12 weeks followed by a wash-out observation period of 4 weeks. There was a statistically significant difference in PASI reduction between M-treatment side (5.92 ±  2.49) and C-treatment side (1.08  ±  1.0) (*P* <  0.001). The authors concluded that vitamin B_12_ ointment will represent a new concrete therapy option and should be considered in the update of therapeutic algorithm for the treatment of psoriasis.

### Quercetin

Quercetin is one of the important bioflavonoids present in more than 20 plant materials and is known for its anti-inflammatory and antiatherosclerotic activities [134]. Quercetin is effective in decreasing serum Hcy level in high methionine-fed rats and one of possible mechanisms is associated with increased transsulfuration of Hcy [135,136]. The exposure of rats to Hcy leads to OS. Administration of quercetin might attenuate oxidative damage induced by Hcy or have a protective effect against it [137].

Using Perry’s scientific mouse tail model, carageenan induced pleurisy in mice and HaCaT cells as experimental models, Vijayalakshmi et al. [138] found that quercetin shows significant orthokeratosis, anti-inflammatory, and maximum antiproliferant activities. The authors concluded that quercetin is promising for further investigations to prove its anti-psoriatic activity. Chen et al. [139] found that quercetin significantly reduces the PASI scores, decrease the temperature of the psoriasis-like lesions, and ameliorates the deteriorating histopathology in imiquimod (IMQ)-induced mice. Moreover, quercetin effectively attenuates the levels of TNF-α, IL-6 and IL-17 in serum and decreases the OS markers in skin tissue in IMQ-induced mice. The mechanism may be associated with the down-regulation of NF-κB pathway. The authors concluded that quercetin has appreciable anti-psoriasis effects in IMQ-induced mice, and has the potential for further development as a candidate for treatment of psoriasis.

### H_2_S donors

H_2_S may play a protective role in the pathogenesis of psoriasis [111]. In the literature, H_2_S may represent an alternative for psoriasis, because it greatly reduced symptoms of a psoriasis-like skin model [140]. H_2_S-releasing agents may be promising therapeutics for psoriasis [111].

Recently, many studies have been carried out with the aim of selecting compounds that can deliver H_2_S to target tissues. These essentially fall into three categories: sulphide salts, naturally occurring compounds, and synthetic H_2_S donors [103]. One possible approach for the therapeutic administration of H_2_S is represented by molecules capable of releasing it in a slow and controlled manner, thus mimicking a physiological level [141]. Fast and uncontrollable H_2_S release can cause severe problems and sometimes can even be lethal [142]. Studies on some of the H_2_S donors regarding their potential use for treatment of psoriasis have been reported.

#### Sulforaphane (SFN)

SFN is an isothiocyanate compound from broccoli (*Brassica oleracea*). The purported health benefits of isothiocyanates are quite similar to those that are attributed to H_2_S or other H_2_S-donating drugs [103]. It was found that a large amount of H_2_S is released when SFN is added into cell culture medium or mixed with mouse liver homogenates, respectively [143]. It was observed that SFN acts as a slow-releasing H_2_S donor supported by several findings [144]. SFN reduced neuropathic pain in mice by releasing H_2_ S [145]. Yehuda et al. [146] found that SFN contributed to the prevention of inflammation development and reduced ongoing inflammation by downregulating lipopolysaccharide (LPS)-induced mRNA expression of the psoriasis-related cytokines IL-12/23p40, TNF-α, and IL-6 in human macrophage-like cells. Moreover, 3/8 of the SFN-treated psoriasiform severe-combined immunodeficient mice recovered partially or entirely from the psoriasiform process. Results from these models indicate the potential of SFN as biological agent in the therapy of psoriasis.

#### N-acetyl cysteine (NAC)

The well-known ability of cysteine to mimic H_2_S effects, presumably by providing additional H_2_S, has led to the evaluation of a number of cysteine analogs as potential substrates for endogenous cysteine-metabolizing enzymes. NAC is one of these molecules [103]. NAC-derived cysteine is desulfurated to generate H_2_S [147]. Supplement with NAC can boost endogenous production of H_2_S by CSE [148]. Increased neutrophil extracellular trap (NET) formation is seen in psoriasis. In psoriasis, the contents of NETs, namely the antimicrobial peptides and the self-DNA, are able to induce IFN-α production from the plasmacytoid dendritic cells. In addition to anti-IFN-α therapies, other novel agents, such as NAC, target NETs. Treatment of neutrophils with NAC blocks ROS and NET formation *in vitro* [149]. In primary human keratinocytes used as a model of inflammatory skin disease and psoriasis, NAC attenuates the IFN-α-induced production of cytokines, suggesting that NAC should be considered as part of effective therapy for the treatment of inflammatory skin diseases, including psoriasis [150].

#### GYY4137

GYY4137 (morpholin-4-ium 4 methoxyphenyl(morpholino) phosphinodithioate) is a synthetic H_2_S donor. It releases H_2_S when dissolved [103]. GYY4137 is generally regarded as a slow-releasing H_2_S donor [151]. Injection of GYY4137 into rats increases plasma H_2_S [103]. Merighi et al. [152] found that application of GYY4137 as H_2_S donor on human keratinocytes significantly enhances nitric oxide (NO) production. The increment in NO down-regulates extracellular signal-regulated kinase 1/2 activation thereby resulting in the decrease of vascular endothelial growth factor (VEGF) release. The authors suggest that GYY4137 may be promising therapeutics for chronic inflammatory disorders of the skin, i.e. psoriasis, in which NO increases as well as anti-VEGF treatments have been suggested to be novel effective approaches.

As we have mentioned above, H_2_S donor may also have a Hcy lowering effect. Until recently, the pharmacological treatments for HHcy have primarily focused upon the supplementation of folic acid and other B vitamins. Although these treatments have been effective at decreasing Hcy levels, improving therapeutic options for treatment of HHcy is still an ongoing effort [6]. The evidence that some H_2_S donors can lower the levels of Hcy may be of pharmacological interest, connecting to the HHcy status in psoriasis.

## Conclusion

According to the literature data including a systematic review and meta-analysis, patients with psoriasis have a significantly higher serum Hcy level and a higher prevalence of HHcy. In psoriasis, insufficient amounts of folic acid and vitamin B_12_ can be a cause of HHcy, and smoking, alcohol consumption and obesity are associated factors.

Hcy may promote the immuno-inflammatory process in the pathogenesis of psoriasis. As a activator, Hcy can facilitate the overactivation of Th1 and Th17 cells. HHcy can also suppress the function of Tregs. HHcy may contribute to the formation of psoriatic lesions by enhancing the activity and migration of neutrophils. The effect of Hcy enhancing the production of the pro-inflammatory cytokines over-expressed in psoriasis suggests a possible role of Hcy in the development of psoriasis by driving the immuno-inflammatory process. Hcy can induce NF-κB activation. This may contribute to the immunopathogenesis of psoriasis. There may be a link between the OS state in psoriasis and the effect of HHcy. H_2_S may play a protective role in the pathogenesis of psoriasis and the deficiency of H_2_S in psoriasis may be caused by HHcy.

As folic acid and vitamin B_12_ can lower Hcy in human body, it is theoretically reasonable to use folic acid and/or vitamin B_12_ to treat psoriasis as a HHcy-related disease. However, effectiveness of systemic administration of these drugs on psoriasis has not been established. On the other hand, systemic folinate calcium, a folic acid derivative, and topical vitamin B12 have found to be effective in treating psoriasis. Such inconsistency should be further studied. A plant-derived compound quercetin which also can lower Hcy levels has been found to have anti-psoriatic effect in animal models. H_2_S donor can increase H_2_S and reduce Hcy. Preclinical studies have indicated the potential of some H_2_S donors as therapeutic agents for treatment of psoriasis in the future.

Investigation on the role of Hcy in the pathogenesis of psoriasis may shed light on new insight into the pathogenic mechanisms in the disease, opening a path for future researches. Data suggest that Hcy can be a potential therapeutic target for treatment of psoriasis.

## Abbreviations

CBS: cystathionine β-synthase
CSE: cystathionine γ-lyase
DC: dendritic cell
Hcy: homocysteine
HHcy: hyperhomocysteinaemia
MMP: matrix metalloproteinase
MTX: methotrexate
NAC: N-acetyl cysteine
NET: neutrophil extracellular trap
NF-κB: nuclear factor κB
NO: nitric oxide
OS: Oxidative stress
PASI: psoriasis area and severity index
PsA: psoriatic arthritis
RA: rheumatoid arthritis
ROS: reactive oxygen species
SAH: S-adenosyl-L-homocysteine
SAM: S-adenosyl-L-methionine
SFN: Sulforaphane
Treg: regulatory T cell
VEGF: vascular endothelial growth factor

## References

[1] Nestle F.O., Kaplan D.H. and Barker J. (2009) Psoriasis. N. Engl. J. Med. 361, 496–509, Review 10.1056/NEJMra0804595 19641206

[2] Hawkes J.E., Chan T.C. and Krueger J.G. (2017) Psoriasis pathogenesis and the development of novel targeted immune therapies. J. Allergy Clin. Immunol. 140, 645–653, Review. PubMed Central PMCID: PMC5600287 10.1016/j.jaci.2017.07.004 28887948 PMC5600287

[3] Lockshin B., Balagula Y. and Merola J.F. (2018) Interleukin 17, inflammation, and cardiovascular risk in patients with psoriasis. J. Am. Acad. Dermatol. 79, 345–352, Epub 2018 Mar 2. Review 10.1016/j.jaad.2018.02.040 29477740

[4] Ganguly P. and Alam S.F. (2015) Role of homocysteine in the development of cardiovascular disease. Nutr. J. 14, 6, Review. PubMed Central PMCID: PMC4326479 10.1186/1475-2891-14-6 25577237 PMC4326479

[5] Moretti R. and Caruso P. (2019) The Controversial Role of Homocysteine in Neurology: From Labs to Clinical Practice. Int. J. Mol. Sci. 20, E231, Review. PubMed Central PMCID: PMC6337226 10.3390/ijms20010231 30626145 PMC6337226

[6] Kumar A., Palfrey H.A., Pathak R., Kadowitz P.J., Gettys T.W. and Murthy S.N. (2017) The metabolism and significance of homocysteine in nutrition and health. Nutr. Metab. (Lond) 14, 78, Review. PubMed Central PMCID: PMC5741875 10.1186/s12986-017-0233-z 29299040 PMC5741875

[7] Fu Y., Wang X. and Kong W. (2018) Hyperhomocysteinaemia and vascular injury: advances in mechanisms and drug targets. Br. J. Pharmacol. 175, 1173–1189, Epub 2017 Sep 22. Review. PubMed Central PMCID: PMC5867019 10.1111/bph.13988 28836260 PMC5867019

[8] Refsum H., Helland S. and Ueland P.M. (1989) Fasting plasma homocysteine as a sensitive parameter of antifolate effect: a study of psoriasis patients receiving low-dose methotrexate treatment. Clin. Pharmacol. Ther. 46, 510–520 10.1038/clpt.1989.179 2582708

[9] Kural B.V., Orem A., Cimşit G., Uydu H.A., Yandi Y.E. and Alver A. (2003) Plasma homocysteine and its relationships with atherothrombotic markers in psoriatic patients. Clin. Chim. Acta 332, 23–30 10.1016/S0009-8981(03)00082-2 12763276

[10] Malerba M., Gisondi P., Radaeli A., Sala R., Calzavara Pinton P.G. and Girolomoni G. (2006) Plasma homocysteine and folate levels in patients with chronic plaque psoriasis. Br. J. Dermatol. 155, 1165–1169, Erratum in: Br J Dermatol. 2007 Feb; 156: 410 10.1111/j.1365-2133.2006.07503.x 17107384

[11] Karabudak O., Ulusoy R.E., Erikci A.A., Solmazgul E., Dogan B. and Harmanyeri Y. (2008) Inflammation and hypercoagulable state in adult psoriatic men. Acta Derm. Venereol. 88, 337–340 18709301 10.2340/00015555-0456

[12] Brazzelli V., Grasso V., Fornara L., Moggio E., Gamba G., Villani S. et al. (2010) Homocysteine, vitamin B12 and folic acid levels in psoriatic patients and correlation with disease severity. Int. J. Immunopathol. Pharmacol. 23, 911–916 10.1177/039463201002300327 20943063

[13] Tobin A.M., Hughes R., Hand E.B., Leong T., Graham I.M. and Kirby B. (2011) Homocysteine status and cardiovascular risk factors in patients with psoriasis: a case-control study. Clin. Exp. Dermatol. 36, 19–23 10.1111/j.1365-2230.2010.03877.x 20545954

[14] Bilgiç Ö, Altınyazar H.C., Baran H. and Ünlü A. (2015) Serum homocysteine, asymmetric dimethyl arginine (ADMA) and other arginine-NO pathway metabolite levels in patients with psoriasis. Arch. Dermatol. Res. 307, 439–444, Epub 2015 Feb 24 10.1007/s00403-015-1553-3 25708188

[15] Giannoni M., Consales V., Campanati A., Ganzetti G., Giuliodori K., Postacchini V. et al. (2015) Homocysteine plasma levels in psoriasis patients: our experience and review of the literature. J. Eur. Acad. Dermatol. Venereol. 29, 1781–1785, Epub 2015 Mar 23 10.1111/jdv.13023 25809089

[16] Khatri G., Mahajan V.K., Mehta K.S., Sharma K.K., Bhushan S. and Chauhan P.S. (2016) Serum levels of homocystiene, vitamin B_12_ and folic acid in Indian patients with psoriasis: results of a pilot study. Our Dermatol Online 7, 276–279 10.7241/ourd.20163.74

[17] Das M., Dawn I., Sarkar S. and Das K. (2017) Plasma homocysteine levels in patients with Psoriasis. Asian J. Med. Sci. 8, 4–7 10.3126/ajms.v8i5.17136

[18] Shalaby M.E., Aref M.I. and Gohar A.M.I. (2017) Homocysteine Serum Status in Patients with Psoriasis Vulgaris. N. Y. Sci. J. 10, 29–32

[19] Cakmak S.K., Gül U., Kiliç C., Gönül M., Soylu S. and Kiliç A. (2009) Homocysteine, vitamin B12 and folic acid levels in psoriasis patients. J. Eur. Acad. Dermatol. Venereol. 23, 300–303, Epub 2008 Dec 18 10.1111/j.1468-3083.2008.03024.x 19207655

[20] Uslu M., Karul A., Gokbulut C., Kozaci D., Savk E., Karaman G. et al. (2013) Investigation of the plasma homocysteine levels in patients with psoriasis. J. Am. Acad. Dermatol. 68, AB204

[21] Erturan I., Köroğlu B.K., Adiloğlu A., Ceyhan A.M., Akkaya V.B., Tamer N. et al. (2014) Evaluation of serum sCD40L and homocysteine levels with subclinical atherosclerosis indicators in patients with psoriasis: a pilot study. Int. J. Dermatol. 53, 503–509 10.1111/ijd.12397 24673360

[22] Akcali C., Buyukcelik B., Kirtak N. and Inaloz S. (2014) Clinical and laboratory parameters associated with metabolic syndrome in Turkish patients with psoriasis. J. Int. Med. Res. 42, 386–394, Epub 2014 Jan 20 10.1177/0300060513502891 24445696

[23] Tsai T.Y., Yen H. and Huang Y.C. (2019) Serum homocysteine, folate and vitamin B(12) levels in patients with psoriasis: a systematic review and meta-analysis. Br. J. Dermatol. 180, 382–389, Epub 2018 Sep 12 10.1111/bjd.17034 30074615

[24] Kim J., Kim H., Roh H. and Kwon Y. (2018) Causes of hyperhomocysteinemia and its pathological significance. Arch. Pharm. Res. 41, 372–383, Epub 2018 Mar 19. Review 10.1007/s12272-018-1016-4 29552692

[25] Fry L., Macdonald A., Almeyda J., Griffin C.J. and Hoffbrand A.V. (1971) The mechanism of folate deficiency in psoriasis. Br. J. Dermatol. 84, 539–544 10.1111/j.1365-2133.1971.tb02543.x 5557509

[26] Touraine R., Revuz J., Zittoun J., Jarret J. and Tulliez M. (1973) Study of folate in psoriasis: blood levels, intestinal absorption and cutaneous loss. Br. J. Dermatol. 89, 335–341 10.1111/j.1365-2133.1973.tb02987.x 4759946

[27] O’Callaghan P., Meleady R., Fitzgerald T., Graham I. and European COMAC group (2002) Smoking and plasma homocysteine. Eur. Heart J. 23, 1580–1586 10.1053/euhj.2002.3172 12323157

[28] Chrysohoou C., Panagiotakos D.B., Pitsavos C., Zeimbekis A., Zampelas A., Papademetriou L. et al. (2004) The associations between smoking, physical activity, dietary habits and plasma homocysteine levels in cardiovascular disease-free people: the ‘ATTICA’ study. Vasc. Med. 9, 117–123 10.1191/1358863x04vm542oa 15521701

[29] Suriyaprom K., Tungtrongchitr R., Pongpaew P., Phonrat B., Harnroongroj T., Vudhivai N. et al. (2005) Homocysteine and vitamin status in healthy Thai smokers. J. Nutr. Environ. Med. 15, 9–21 10.1080/13590840500220197

[30] Haj Mouhamed D., Ezzaher A., Neffati F., Douki W. and Najjar M.F. (2011) Effect of cigarette smoking on plasma homocysteine concentrations. Clin. Chem. Lab. Med. 49, 479–483, Epub 2010 Dec 14 21143017 10.1515/CCLM.2011.062

[31] Richer V., Roubille C., Fleming P., Starnino T., McCourt C., McFarlane A. et al. (2016) Psoriasis and Smoking: A Systematic Literature Review and Meta-Analysis With Qualitative Analysis of Effect of Smoking on Psoriasis Severity. J. Cutan. Med. Surg. 20, 221–227, Epub 2015 Nov 9. Review 10.1177/1203475415616073 26553732

[32] Hultberg B., Berglund M., Andersson A. and Frank A. (1993) Elevated plasma homocysteine in alcoholics. Alcohol Clin. Exp. Res. 17, 687–689 10.1111/j.1530-0277.1993.tb00820.x 8392819

[33] Cravo M.L., Glória L.M., Selhub J., Nadeau M.R., Camilo M.E., Resende M.P. et al. (1996) Hyperhomocysteinemia in chronic alcoholism: correlation with folate, vitamin B-12, and vitamin B-6 status. Am. J. Clin. Nutr. 63, 220–224 10.1093/ajcn/63.2.220 8561063

[34] van der Gaag M.S., Ubbink J.B., Sillanaukee P., Nikkari S. and Hendriks H.F. (2000) Effect of consumption of red wine, spirits, and beer on serum homocysteine. Lancet 355, 1522 10.1016/S0140-6736(00)02172-3 10801179

[35] Koehler K.M., Baumgartner R.N., Garry P.J., Allen R.H., Stabler S.P. and Rimm E.B. (2001) Association of folate intake and serum homocysteine in elderly persons according to vitamin supplementation and alcohol use. Am. J. Clin. Nutr. 73, 628–637 10.1093/ajcn/73.3.628 11237942

[36] Bleich S., Bleich K., Kropp S., Bittermann H.J., Degner D., Sperling W. et al. (2001) Moderate alcohol consumption in social drinkers raises plasma homocysteine levels: a contradiction to the ‘French Paradox’? Alcohol Alcohol. 36, 189–192 10.1093/alcalc/36.3.189 11373253

[37] Pitsavos C., Panagiotakos D.B., Kontogianni M.D., Chrysohoou C., Chloptsios Y., Zampelas A. et al. (2004) The J-shape association of ethanol intake with total homocysteine concentrations: the ATTICA study. Nutr. Metab. (Lond) 1, 9, PubMed Central PMCID: PMC526386 10.1186/1743-7075-1-9 15507131 PMC526386

[38] Sakuta H. and Suzuki T. (2005) Alcohol consumption and plasma homocysteine. Alcohol 37, 73–77 10.1016/j.alcohol.2005.12.005 16584970

[39] Bleich S., Carl M., Bayerlein K., Reulbach U., Biermann T., Hillemacher T. et al. (2005) Evidence of increased homocysteine levels in alcoholism: the Franconian alcoholism research studies (FARS). Alcohol Clin. Exp. Res. 29, 334–336 10.1097/01.ALC.0000156083.91214.59 15770107

[40] Gibson A., Woodside J.V., Young I.S., Sharpe P.C., Mercer C., Patterson C.C. et al. (2008) Alcohol increases homocysteine and reduces B vitamin concentration in healthy male volunteers–a randomized, crossover intervention study. QJM 101, 881–887, Epub 2008 Sep 12. PubMed Central PMCID: PMC2572692 10.1093/qjmed/hcn112 18790817 PMC2572692

[41] Brenaut E., Horreau C., Pouplard C., Barnetche T., Paul C., Richard M.A. et al. (2013) Alcohol consumption and psoriasis: a systematic literature review. J. Eur. Acad. Dermatol. Venereol. 27, 30–35, Review 10.1111/jdv.12164 23845150

[42] Armstrong A.W., Harskamp C.T. and Armstrong E.J. (2012) The association between psoriasis and obesity: a systematic review and meta-analysis of observational studies. Nutr. Diabetes 2, e54, PubMed Central PMCID: PMC3542430 10.1038/nutd.2012.26 23208415 PMC3542430

[43] Aune D., Snekvik I., Schlesinger S., Norat T., Riboli E. and Vatten L.J. (2018) Body mass index, abdominal fatness, weight gain and the risk of psoriasis: a systematic review and dose-response meta-analysis of prospective studies. Eur. J. Epidemiol. 33, 1163–1178, Epub 2018 Apr 21. PubMed Central PMCID: PMC6290660 10.1007/s10654-018-0366-z 29680995 PMC6290660

[44] Marchesini G., Manini R., Bianchi G., Sassi S., Natale S., Chierici S. et al. (2002) Homocysteine and psychological traits: a study in obesity. Nutrition 18, 403–407 10.1016/S0899-9007(01)00803-6 11985945

[45] Tungtrongchitr R., Pongpaew P., Tongboonchoo C., Vudhivai N., Changbumrung S., Tungtrongchitr A. et al. (2003) Serum homocysteine, B12 and folic acid concentration in Thai overweight and obese subjects. Int. J. Vitam. Nutr. Res. 73, 8–14 10.1024/0300-9831.73.1.8 12690905

[46] Konukoğlu D., Serin O., Ercan M. and Turhan M.S. (2003) Plasma homocysteine levels in obese and non-obese subjects with or without hypertension; its relationship with oxidative stress and copper. Clin. Biochem. 36, 405–408 10.1016/S0009-9120(03)00059-6 12849875

[47] Sanlier N. and Yabanci N. (2007) Relationship between body mass index, lipids and homocysteine levels in university students. J. Pak. Med. Assoc. 57, 491–495 17990423

[48] Karatela R.A. and Sainani G.S. (2009) Plasma homocysteine in obese, overweight and normal weight hypertensives and normotensives. Indian Heart J. 61, 156–159 20039500

[49] Kumar K.J., Saldanha K., Sushma K., Murthy D.S. and Vishwanath P. (2017) A Prospective Study of Homocysteine and its relation to Body Mass Index and Lipid Profile in School Children. Indian Pediatr. 54, 935–937, Epub 2017 Aug 24 10.1007/s13312-017-1185-0 28849772

[50] Rendon A. and Schäkel K. (2019) Psoriasis Pathogenesis and Treatment. Int. J. Mol. Sci. 20, E1475, Review. PubMed Central PMCID: PMC6471628 10.3390/ijms20061475 30909615 PMC6471628

[51] Johnson-Huang L.M., Lowes M.A. and Krueger J.G. (2012) Putting together the psoriasis puzzle: an update on developing targeted therapies. Dis. Model Mech. 5, 423–433, Review. PubMed Central PMCID: PMC3380706 10.1242/dmm.009092 22730473 PMC3380706

[52] Rutault K., Alderman C., Chain B.M. and Katz D.R. (1999) Reactive oxygen species activate human peripheral blood dendritic cells. Free Radic. Biol. Med. 26, 232–238 10.1016/S0891-5849(98)00194-4 9890657

[53] Batal I., Azzi J., Mounayar M., Abdoli R., Moore R., Lee J.Y. et al. (2014) The mechanisms of up-regulation of dendritic cell activity by oxidative stress. J. Leukoc. Biol. 96, 283–293, Epub 2014 Mar 27. PubMed Central PMCID: PMC4101089 10.1189/jlb.3A0113-033RR 24676276 PMC4101089

[54] Škovierová H., Vidomanová E., Mahmood S., Sopková J., Drgová A., Červeňová T. et al. (2016) The Molecular and Cellular Effect of Homocysteine Metabolism Imbalance on Human Health. Int. J. Mol. Sci. 17, E1733, Review. PubMed Central PMCID: PMC5085763 10.3390/ijms17101733 27775595 PMC5085763

[55] Sawai A., Ohshige K., Kura N. and Tochikubo O. (2008) Influence of mental stress on the plasma homocysteine level and blood pressure change in young men. Clin. Exp. Hypertens. 30, 233–241 10.1080/10641960802068725 18425703

[56] Weigle N. and McBane S. (2013) Psoriasis. Am. Fam. Physician 87, 626–633, Review 23668525

[57] Lin X. and Huang T. (2016) Impact of pregnancy and oestrogen on psoriasis and potential therapeutic use of selective oestrogen receptor modulators for psoriasis. J. Eur. Acad. Dermatol. Venereol. 30, 1085–1091, Epub 2016 Apr 13. Review 10.1111/jdv.13661 27072912

[58] Hague W.M. (2003) Homocysteine and pregnancy. Best Pract. Res. Clin. Obstet. Gynaecol. 17, 459–469, Review 10.1016/S1521-6934(03)00009-9 12787538

[59] Barrea L., Balato N., Di Somma C., Macchia P.E., Napolitano M., Savanelli M.C. et al. (2015) Nutrition and psoriasis: is there any association between the severity of the disease and adherence to the Mediterranean diet? J. Transl. Med. 13, 18, PubMed Central PMCID:PMC4316654 10.1186/s12967-014-0372-1 25622660 PMC4316654

[60] Foscolou A., Rallidis L.S., Tsirebolos G., Critselis E., Katsimardos A., Drosatos A. et al. (2019) The association between homocysteine levels, Mediterranean diet and cardiovascular disease: a case-control study. Int. J. Food Sci. Nutr. 70, 603–611, Epub 2018 Dec 2 10.1080/09637486.2018.1547688 30501542

[61] Zhang Q., Zeng X., Guo J. and Wang X. (2002) Oxidant stress mechanism of homocysteine potentiating Con A-induced proliferation in murine splenic T lymphocytes. Cardiovasc. Res. 53, 1035–1042 10.1016/S0008-6363(01)00541-7 11922914

[62] Diani M., Altomare G. and Reali E. (2016) T Helper Cell Subsets in Clinical Manifestations of Psoriasis. J. Immunol. Res. 2016, 7692024, Epub 2016 Aug 10. Review. PubMed Central PMCID: PMC4995329 10.1155/2016/7692024 27595115 PMC4995329

[63] Cai Y., Fleming C. and Yan J. (2012) New insights of T cells in the pathogenesis of psoriasis. Cell Mol. Immunol. 9, 302–309, Epub 2012 Jun 18. Review. PubMed Central PMCID: PMC4132586 10.1038/cmi.2012.15 22705915 PMC4132586

[64] Dawson H., Collins G., Pyle R., Deep-Dixit V. and Taub D.D. (2004) The immunoregulatory effects of homocysteine and its intermediates on T-lymphocyte function. Mech. Ageing Dev. 125, 107–110, Review 10.1016/j.mad.2003.11.013 15037011

[65] Dai J. and Wang X. (2007) Immunoregulatory effects of homocysteine on cardiovascular diseases. Sheng Li Xue Bao 59, 585–592 17940698

[66] Feng J., Zhang Z., Kong W., Liu B., Xu Q. and Wang X. (2009) Regulatory T cells ameliorate hyperhomocysteinaemia-accelerated atherosclerosis in apoE-/- mice. Cardiovasc. Res. 84, 155–163, Epub 2009 Jun 5 10.1093/cvr/cvp182 19502284

[67] Ma K., Lv S., Liu B., Liu Z., Luo Y., Kong W. et al. (2013) CTLA4-IgG ameliorates homocysteine-accelerated atherosclerosis by inhibiting T-cell overactivation in apoE(-/-) mice. Cardiovasc. Res. 97, 349–359, Epub 2012 Oct 31 10.1093/cvr/cvs330 23118130

[68] Gao X., Li J. and Chen M. (2018) Effect of Homocysteine on the Differentiation of CD4(+) T Cells into Th17 Cells. Dig. Dis. Sci. 63, 3339–3347, Epub 2018 Jul 4 10.1007/s10620-018-5177-2 29974377

[69] Mattozzi C., Salvi M., D’Epiro S., Giancristoforo S., Macaluso L., Luci C. et al. (2013) Importance of regulatory T cells in the pathogenesis of psoriasis: review of the literature. Dermatology 227, 134–145, Epub 2013 Sep 14. Review 10.1159/000353398 24051528

[70] Flammer A.J. and Ruschitzka F. (2012) Psoriasis and atherosclerosis: two plaques, one syndrome? Eur. Heart J. 33, 1989–1991, Epub 2011 Nov 21 10.1093/eurheartj/ehr425 22108835

[71] Chiricozzi A., Romanelli P., Volpe E., Borsellino G. and Romanelli M. (2018) Scanning the Immunopathogenesis of Psoriasis. Int. J. Mol. Sci. 19, E179, Review. PubMed Central PMCID: PMC5796128 10.3390/ijms19010179 29316717 PMC5796128

[72] Alvarez-Maqueda M., El Bekay R., Monteseirín J., Alba G., Chacón P., Vega A. et al. (2004) Homocysteine enhances superoxide anion release and NADPH oxidase assembly by human neutrophils. Effects on MAPK activation and neutrophil migration. Atherosclerosis. 172, 229–238 10.1016/j.atherosclerosis.2003.11.005 15019532

[73] Nedoszytko B., Sokołowska-Wojdyło M., Ruckemann-Dziurdzińska K., Roszkiewicz J. and Nowicki R.J. (2014) Chemokines and cytokines network in the pathogenesis of the inflammatory skin diseases: atopic dermatitis, psoriasis and skin mastocytosis. Postepy Dermatol. Alergol. 31, 84–91, Epub 2014 Apr 22. Review. PubMed Central PMCID: PMC4112246 10.5114/pdia.2014.40920 25097473 PMC4112246

[74] Yun J., Kim J.Y., Kim O.Y., Jang Y., Chae J.S., Kwak J.H. et al. (2011) Associations of plasma homocysteine level with brachial-ankle pulse wave velocity, LDL atherogenicity, and inflammation profile in healthy men. Nutr. Metab. Cardiovasc. Dis. 21, 136–143, Epub 2009 Oct 23 10.1016/j.numecd.2009.08.003 19854035

[75] Fefelova E.V., Tereshkov P.P., Dutov A.A. and Tsybikov N.N. (2015) Lymphocyte Subpopulations and Cytokine Levels in Experimental Hyperhomocysteinemia. Bull. Exp. Biol. Med. 159, 358–360, Epub 2015 Jul 28 10.1007/s10517-015-2962-1 26212809

[76] da Cunha A.A., Ferreira A.G. and Wyse A.T. (2010) Increased inflammatory markers in brain and blood of rats subjected to acute homocysteine administration. Metab. Brain Dis. 25, 199–206, Epub 2010 Apr 28 10.1007/s11011-010-9188-8 20424906

[77] da Cunha A.A., Ferreira A.G., Loureiro S.O., da Cunha M.J., Schmitz F., Netto C.A. et al. (2012) Chronic hyperhomocysteinemia increases inflammatory markers in hippocampus and serum of rats. Neurochem. Res. 37, 1660–1669, Epub 2012 Apr 8 10.1007/s11064-012-0769-2 22484967

[78] Scherer E.B., Loureiro S.O., Vuaden F.C., da Cunha A.A., Schmitz F., Kolling J. et al. (2014) Mild hyperhomocysteinemia increases brain acetylcholinesterase and proinflammatory cytokine levels in different tissues. Mol. Neurobiol. 50, 589–596, Epub 2014 Mar 5 10.1007/s12035-014-8660-6 24590316

[79] Zhang D., Fang P., Jiang X., Nelson J., Moore J.K., Kruger W.D. et al. (2012) Severe hyperhomocysteinemia promotes bone marrow-derived and resident inflammatory monocyte differentiation and atherosclerosis in LDLr/CBS-deficient mice. Circ. Res. 111, 37–49, Epub 2012 May 24. PubMed Central PMCID: PMC3412115 10.1161/CIRCRESAHA.112.269472 22628578 PMC3412115

[80] Aso Y., Okumura K., Takebayashi K., Wakabayashi S. and Inukai T. (2003) Relationships of plasma interleukin-18 concentrations to hyperhomocysteinemia and carotid intimal-media wall thickness in patients with type 2 diabetes. Diabetes Care 26, 2622–2627 10.2337/diacare.26.9.2622 12941729

[81] McLachlan C.S., Chua W.C., Wong P.T., Kah T.L., Chen C. and El Oakley R.M. (2005) Homocysteine is positively associated with cytokine IL-18 plasma levels in coronary artery bypass surgery patients. Biofactors 23, 69–73 10.1002/biof.5520230202 16179748

[82] Tso T.K., Huang W.N., Huang H.Y. and Chang C.K. (2006) Relationship of plasma interleukin-18 concentrations to traditional and non-traditional cardiovascular risk factors in patients with systemic lupus erythematosus. Rheumatology (Oxford) 45, 1148–1153, Epub 2006 Mar 9 10.1093/rheumatology/kel082 16527878

[83] Wang R., Wang Y., Mu N., Lou X., Li W., Chen Y. et al. (2017) Activation of NLRP3 inflammasomes contributes to hyperhomocysteinemia-aggravated inflammation and atherosclerosis in apoE-deficient mice. Lab. Invest. 97, 922–934, Epub 2017 Apr 10. PubMed Central PMCID: PMC5537437 10.1038/labinvest.2017.30 28394319 PMC5537437

[84] Dalal S., Parkin S.M., Homer-Vanniasinkam S. and Nicolaou A. (2003) Effect of homocysteine on cytokine production by human endothelial cells and monocytes. Ann. Clin. Biochem. 40, 534–541 10.1258/000456303322326452 14503991

[85] Su S.J., Huang L.W., Pai L.S., Liu H.W. and Chang K.L. (2005) Homocysteine at pathophysiologic concentrations activates human monocyte and induces cytokine expression and inhibits macrophage migration inhibitory factor expression. Nutrition 21, 994–1002 10.1016/j.nut.2005.01.011 16157236

[86] Zeng X.K., Remick D.G. and Wang X. (2004) Homocysteine induces production of monocyte chemoattractant protein-1 and interleukin-8 in cultured human whole blood. Acta Pharmacol. Sin. 25, 1419–1425 15525462

[87] Georgescu S.R., Tampa M., Caruntu C., Sarbu M.I., Mitran C.I., Mitran M.I. et al. (2019) Advances in Understanding the Immunological Pathways in Psoriasis. Int. J. Mol. Sci. 20, E739, Review. PubMed Central PMCID: PMC6387410 10.3390/ijms20030739 30744173 PMC6387410

[88] Owczarczyk-Saczonek A., Czerwińska J. and Placek W. (2018) The role of regulatory T cells and anti-inflammatory cytokines in psoriasis. Acta Dermatovenerol Alp Pannonica Adriat 27, 17–23, Review 29589640

[89] Li T., Gu M., Liu P., Liu Y., Guo J., Zhang W. et al. (2018) Clinical Significance of Decreased Interleukin-35 Expression in Patients with Psoriasis. Microbiol. Immunol. [Epub ahead of print] 10.1111/1348-0421.12605 29802736

[90] Głażewska E.K., Niczyporuk M., Ławicki S., Szmitkowski M., Donejko M., Zajkowska M. et al. (2017) Narrowband ultraviolet B light treatment changes plasma concentrations of MMP-3, MMP-9 and TIMP-3 in psoriatic patients. Ther. Clin. Risk Manag. 13, 575–582, eCollection 2017. PubMed Central PMCID: PMC5414720 10.2147/TCRM.S125595 28490884 PMC5414720

[91] Holven K.B., Halvorsen B., Bjerkeli V., Damås J.K., Retterstøl K., Mørkrid L. et al. (2006) Impaired inhibitory effect of interleukin-10 on the balance between matrix metalloproteinase-9 and its inhibitor in mononuclear cells from hyperhomocysteinemic subjects. Stroke 37, 1731–1736, Epub 2006 May 25 10.1161/01.STR.0000226465.84561.cb 16728689

[92] Flannigan K.L., Agbor T.A., Blackler R.W., Kim J.J., Khan W.I., Verdu E.F. et al. (2014) Impaired hydrogen sulfide synthesis and IL-10 signaling underlie hyperhomocysteinemia-associated exacerbation of colitis. Proc. Natl. Acad. Sci. U.S.A. 111, 13559–13564, Epub 2014 Sep 3. PubMed Central PMCID: PMC4169975 10.1073/pnas.1413390111 25187563 PMC4169975

[93] Owczarczyk-Saczonek A., Czerwińska J. and Placek W. (2018) The role of regulatory T cells and anti-inflammatory cytokines in psoriasis. Acta Dermatovenerol Alp Pannonica Adriat 27, 17–23, Review 29589640

[94] Goldminz A.M., Au S.C., Kim N., Gottlieb A.B. and Lizzul P.F. (2013) NF-κB: an essential transcription factor in psoriasis. J. Dermatol. Sci. 69, 89–94, Epub 2012 Nov 14. Review 10.1016/j.jdermsci.2012.11.002 23219896

[95] Wang G., Siow Y.L. and O K. (2000) Homocysteine stimulates nuclear factor kappaB activity and monocyte chemoattractant protein-1 expression in vascular smooth-muscle cells: a possible role for protein kinase C. Biochem. J. 352, 817–826, PubMed Central PMCID: PMC1221522 10.1042/bj3520817 11104691 PMC1221522

[96] Wang G., Siow Y.L. and O K. (2001) Homocysteine induces monocyte chemoattractant protein-1 expression by activating NF-kappaB in THP-1 macrophages. Am. J. Physiol. Heart Circ. Physiol. 280, H2840–H2847 10.1152/ajpheart.2001.280.6.H2840 11356643

[97] Au-Yeung K.K., Woo C.W., Sung F.L., Yip J.C., Siow Y.L. and Karmin O. (2004) Hyperhomocysteinemia activates nuclear factor-kappaB in endothelial cells via oxidative stress. Circ. Res. 94, 28–36, Epub 2003 Nov 20 10.1161/01.RES.0000108264.67601.2C 14630727

[98] Liu B., Ma S., Wang T., Zhao C., Li Y., Yin J. et al. (2016) A novel rat model of heart failure induced by high methionine diet showing evidence of association between hyperhomocysteinemia and activation of NF-kappaB. Am. J. Transl. Res. 8, 117–124, eCollection 2016. PubMed Central PMCID: PMC4759421 27069545 PMC4759421

[99] Moorchung N., Kulaar J.S., Chatterjee M., Vasudevan B., Tripathi T. and Dutta V. (2014) Role of NF-κB in the pathogenesis of psoriasis elucidated by its staining in skin biopsy specimens. Int. J. Dermatol. 53, 570–574, Epub 2013 Apr 28 10.1111/ijd.12050 23621342

[100] Liu T., Zhang L., Joo D. and Sun S.C. (2017) NF-κB signaling in inflammation. Signal Transduct Target Ther. 2, 17023, Epub 2017 Jul 14. PubMed Central PMCID: PMC5661633 10.1038/sigtrans.2017.23 29158945 PMC5661633

[101] Škovierová H., Vidomanová E., Mahmood S., Sopková J., Drgová A., Červeňová T. et al. (2016) The Molecular and Cellular Effect of Homocysteine Metabolism Imbalance on Human Health. Int. J. Mol. Sci. 17, E1733, Review. PubMed Central PMCID: PMC5085763 10.3390/ijms17101733 27775595 PMC5085763

[102] Lin X. and Huang T. (2016) Oxidative stress in psoriasis and potential therapeutic use of antioxidants. Free Radic. Res. 50, 585–595, Epub 2016 Apr 21. Review 10.3109/10715762.2016.1162301 27098416

[103] Kashfi K. and Olson K.R. (2013) Biology and therapeutic potential of hydrogen sulfide and hydrogen sulfide-releasing chimeras. Biochem. Pharmacol. 85, 689–703, Epub 2012 Oct 24. Review. PubMed Central PMCID: PMC3566320 10.1016/j.bcp.2012.10.019 23103569 PMC3566320

[104] Sen U., Mishra P.K., Tyagi N. and Tyagi S.C. (2010) Homocysteine to hydrogen sulfide or hypertension. Cell Biochem. Biophys. 57, 49–58, Review. PubMed Central PMCID: PMC2921842 10.1007/s12013-010-9079-y 20387006 PMC2921842

[105] Chang L., Geng B., Yu F., Zhao J., Jiang H., Du J. et al. (2008) Hydrogen sulfide inhibits myocardial injury induced by homocysteine in rats. Amino Acids 34, 573–585, Epub 2007 Dec 11. Erratum in: Amino Acids. 2008 May; 34: 687 10.1007/s00726-007-0011-8 18071843

[106] Kumar M., Modi M. and Sandhir R. (2017) Hydrogen sulfide attenuates homocysteine-induced cognitive deficits and neurochemical alterations by improving endogenous hydrogen sulfide levels. Biofactors 43, 434–450, Epub 2017 Apr 10 10.1002/biof.1354 28394038

[107] Nath N., Prasad H.K. and Kumar M. (2019) Cerebroprotective effects of hydrogen sulfide in homocysteine-induced neurovascular permeability: Involvement of oxidative stress, arginase, and matrix metalloproteinase-9. J. Cell. Physiol. 234, 3007–3019, Epub 2018 Sep 12 10.1002/jcp.27120 30206943

[108] Sbodio J.I., Snyder S.H. and Paul B.D. (2019) Regulators of the transsulfuration pathway. Br. J. Pharmacol. 176, 583–593, Epub 2018 Aug 23. Review. PubMed Central PMCID: PMC6346075 10.1111/bph.14446 30007014 PMC6346075

[109] Yakovleva O.V., Ziganshina A.R., Dmitrieva S.A., Arslanova A.N., Yakovlev A.V., Minibayeva F.V. et al. (2018) Hydrogen Sulfide Ameliorates Developmental Impairments of Rat Offspring with Prenatal Hyperhomocysteinemia. Oxid. Med. Cell. Longev 2018, 2746873, eCollection 2018. PubMed Central PMCID: PMC6276483 10.1155/2018/2746873 30581528 PMC6276483

[110] Parsanathan R. and Jain S.K. (2018) Hydrogen sulfide increases glutathione biosynthesis, and glucose uptake and utilisation in C(2)C(12) mouse myotubes. Free Radic. Res. 52, 288–303, PubMed Central PMCID: PMC6181233 10.1080/10715762.2018.1431626 29378451 PMC6181233

[111] Alshorafa A.K., Guo Q., Zeng F., Chen M., Tan G., Tang Z. et al. (2012) Psoriasis is associated with low serum levels of hydrogen sulfide, a potential anti-inflammatory molecule. Tohoku J. Exp. Med. 228, 325–332 10.1620/tjem.228.325 23132229

[112] Busse K. and Liao W. (2010) Which Psoriasis Patients Develop Psoriatic Arthritis? Psoriasis Forum 16, 17–25, PubMed Central PMCID: PMC4206220 10.1177/247553031016a00403 25346592 PMC4206220

[113] Queiro R., Morante I., Cabezas I. and Acasuso B. (2016) HLA-B27 and psoriatic disease: a modern view of an old relationship. Rheumatology (Oxford) 55, 221–229, Epub 2015 Aug 19. Review 10.1093/rheumatology/kev296 26289052

[114] Segal R., Baumoehl Y., Elkayam O., Levartovsky D., Litinsky I., Paran D. et al. (2004) Anemia, serum vitamin B12, and folic acid in patients with rheumatoid arthritis, psoriatic arthritis, and systemic lupus erythematosus. Rheumatol. Int. 24, 14–19, Epub 2003 Apr 29 10.1007/s00296-003-0323-2 12720045

[115] Rendon A. and Schäkel K. (2019) Psoriasis Pathogenesis and Treatment. Int. J. Mol. Sci. 20, E1475, Review. PubMed Central PMCID: PMC647162 10.3390/ijms20061475 30909615 PMC6471628

[116] Czarnecka-Operacz M. and Sadowska-Przytocka A. (2014) The possibilities and principles of methotrexate treatment of psoriasis - the updated knowledge. Postepy Dermatol. Alergol. 31, 392–400, Epub 2014 Dec 3. Review. PubMed Central PMCID: PMC4293394 10.5114/pdia.2014.47121 25610355 PMC4293394

[117] Hoekstra M., Haagsma C.J., Doelman C.J. and van de Laar M.A. (2005) Intermittent rises in plasma homocysteine in patients with rheumatoid arthritis treated with higher dose methotrexate. Ann. Rheum. Dis. 64, 141–143, PubMed Central PMCID: PMC1755168 10.1136/ard.2003.019828 15608313 PMC1755168

[118] McDonald I., Connolly M. and Tobin A.M. (2012) A review of psoriasis, a known risk factor for cardiovascular disease and its impact on folate and homocysteine metabolism. J. Nutr. Metab. 2012, 965385, Epub 2012 May 29. PubMed Central PMCID: PMC3368579 10.1155/2012/965385 22690330 PMC3368579

[119] Hornung N., Ellingsen T., Stengaard-Pedersen K. and Poulsen J.H. (2004) Folate, homocysteine, and cobalamin status in patients with rheumatoid arthritis treated with methotrexate, and the effect of low dose folic acid supplement. J. Rheumatol. 31, 2374–2381 15570637

[120] Amor K.T., Ryan C. and Menter A. (2010) The use of cyclosporine in dermatology: part I. J. Am. Acad. Dermatol. 63, 925–946, quiz 947-8. Review 10.1016/j.jaad.2010.02.063 21093659

[121] Arnadottir M., Hultberg B., Vladov V., Nilsson-Ehle P. and Thysell H. (1996) Hyperhomocysteinemia in cyclosporine-treated renal transplant recipients. Transplantation 61, 509–512 10.1097/00007890-199602150-00034 8610370

[122] Akoglu B., Wondra K., Caspary W.F. and Faust D. (2006) Determinants of fasting total serum homocysteine levels in liver transplant recipients. Exp. Clin. Transplant. 4, 462–466 16827644

[123] e Silva Ade S. and da Mota M.P. (2014) Effects of physical activity and training programs on plasma homocysteine levels: a systematic review. Amino Acids 46, 1795–1804, Epub 2014 Apr 26. Review 10.1007/s00726-014-1741-z 24770903

[124] Dawson T.A., Scott K.W. and Merrett J.D. (1978) Trial of folic acid therapy in psoriasis. Ulster Med. J. 47, 100–101, PubMed Central PMCID: PMC2385860 349814 PMC2385860

[125] Aronson P.J. (2017) Cases of psoriasis improved by lowering homocysteine using 4-7 mg folic acid, vitamins B6 and B12 previously worsened using 1-2 mg daily folic acid, B6 and B12 folic acid. J. Transl. Sci. 3, 1–6 10.15761/JTS.1000193

[126] Gisondi P., Fantuzzi F., Malerba M. and Girolomoni G. (2007) Folic acid in general medicine and dermatology. J. Dermatolog. Treat. 18, 138–146, Review 10.1080/09546630701247930 17538801

[127] Claussen D.W. (1997) Common vitamin B complex agents and folic acid: Part IV. Leucovorin calcium (citrovorum factor, folinic acid [wellcovorin]). Gastroenterol Nurs. 20, 107–108, Review 10.1097/00001610-199705000-00009 9238940

[128] Carlesimo M., Mari E., Arcese A., De Angelis F., Palese E., Abruzzese C. et al. (2010) Safety and efficacy of calcium folinate in psoriasis: an observational study. Int. J. Immunopathol. Pharmacol. 23, 649–653 10.1177/039463201002300229 20646362

[129] Baker H. and Comaish J.S. (1962) Is vitamin B12 of value in psoriasis? Br. Med. J. 2, 1729–1730, PubMed Central PMCID: PMC1926971 10.1136/bmj.2.5321.1729 13969145 PMC1926971

[130] Carslaw R.W. and Neill J. (1963) Vitamin B 12 in P psoriasis. Br. Med. J. 1, 611, PubMed Central PMCID: PMC2123545 10.1136/bmj.1.5330.611 14018924 PMC2123545

[131] Stankler L. (1969) The vitamin B12 level in psoriatic skin and serum. Br. J. Dermatol. 81, 911–918 10.1111/j.1365-2133.1969.tb15973.x 5359454

[132] Stücker M., Memmel U., Hoffmann M., Hartung J. and Altmeyer P. (2001) Vitamin B(12) cream containing avocado oil in the therapy of plaque psoriasis. Dermatology 203, 141–147 10.1159/000051729 11586013

[133] Del Duca E., Farnetani F., De Carvalho N., Bottoni U., Pellacani G. and Nisticò S.P. (2017) Superiority of a vitamin B(12)-containing emollient compared to a standard emollient in the maintenance treatment of mild-to-moderate plaque psoriasis. Int. J. Immunopathol. Pharmacol. 30, 439–444, Epub 2017 Oct 19. PubMed Central PMCID: PMC5806803 10.1177/0394632017736674 29048238 PMC5806803

[134] Anand David A.V., Arulmoli R. and Parasuraman S. (2016) Overviews of Biological Importance of Quercetin: A Bioactive Flavonoid. Pharmacogn. Rev. 10, 84–89, Review. PubMed Central PMCID: PMC5214562 10.4103/0973-7847.194044 28082789 PMC5214562

[135] Meng B., Gao W., Wei J., Yang J., Wu J., Pu L. et al. (2013) Quercetin reduces serum homocysteine level in rats fed a methionine-enriched diet. Nutrition 29, 661–666, Epub 2013 Feb 12 10.1016/j.nut.2012.10.012 23410631

[136] Meng B., Gao W., Wei J., Pu L., Tang Z. and Guo C. (2015) Quercetin Increases Hepatic Homocysteine Remethylation and Transsulfuration in Rats Fed a Methionine-Enriched Diet. Biomed. Res. Int. 2015, 815210, Epub 2015 Oct19. PubMed Central PMCID: PMC4629001 10.1155/2015/815210 26558284 PMC4629001

[137] Çelik N., Vurmaz A. and Kahraman A. (2017) Protective effect of quercetin on homocysteine-induced oxidative stress. Nutrition 33, 291–296, Epub 2016 Aug 6 10.1016/j.nut.2016.07.014 27717661

[138] Vijayalakshmi A., Ravichandiran V., Malarkodi V., Nirmala S. and Jayakumari S. (2012) Screening of flavonoid “quercetin” from the rhizome of Smilax china Linn. for anti-psoriatic activity. Asian Pac. J. Trop. Biomed. 2, 269–275, PubMed Central PMCID: PMC3609293 10.1016/S2221-1691(12)60021-5 23569912 PMC3609293

[139] Chen H., Lu C., Liu H., Wang M., Zhao H., Yan Y. et al. (2017) Quercetin ameliorates imiquimod-induced psoriasis-like skin inflammation in mice via the NF-κB pathway. Int. Immunopharmacol. 48, 110–117, Epub 2017 May 10 10.1016/j.intimp.2017.04.022 28499194

[140] Xiao Q., Ying J., Xiang L. and Zhang C. (2018) The biologic effect of hydrogen sulfide and its function in various diseases. Medicine (Baltimore). 97, e13065, Review. PubMed Central PMCID: PMC6221678 10.1097/MD.0000000000013065 30383685 PMC6221678

[141] Giustarini D., Tazzari V., Bassanini I., Rossi R. and Sparatore A. (2018) The new H(2)S-releasing compound ACS94 exerts protective effects through the modulation of thiol homoeostasis. J. Enzyme Inhib. Med. Chem. 33, 1392–1404, PubMed Central PMCID:PMC6127811 10.1080/14756366.2018.1509211 30173573 PMC6127811

[142] Zhao Y., Biggs T.D. and Xian M. (2014) Hydrogen sulfide (H2S) releasing agents: chemistry and biological applications. Chem. Commun. (Camb.) 50, 11788–11805, Review. PubMed Central PMCID: PMC4162744 10.1039/C4CC00968A 25019301 PMC4162744

[143] Pei Y., Wu B., Cao Q., Wu L. and Yang G. (2011) Hydrogen sulfide mediates the anti-survival effect of sulforaphane on human prostate cancer cells. Toxicol. Appl. Pharmacol. 257, 420–428, Epub 2011 Oct 8 10.1016/j.taap.2011.09.026 22005276

[144] Liu M., Wu L., Montaut S. and Yang G. (2016) Hydrogen Sulfide Signaling Axis as a Target for Prostate Cancer Therapeutics. Prostate Cancer 2016, 8108549, Epub 2016 Feb 25. Review. PubMed Central PMCID: PMC478527 10.1155/2016/8108549 27019751 PMC4785274

[145] Lucarini E., Micheli L., Trallori E., Citi V., Martelli A., Testai L. et al. (2018) Effect of glucoraphanin and sulforaphane against chemotherapy-induced neuropathic pain: Kv7 potassium channels modulation by H(2) S release in vivo. Phytother. Res. 32, 2226–2234, Epub 2018 Aug 2 10.1002/ptr.6159 30069944

[146] Yehuda H., Soroka Y., Zlotkin-Frušić M., Gilhar A., Milner Y. and Tamir S. (2012) Isothiocyanates inhibit psoriasis-related proinflammatory factors in human skin. Inflamm. Res. 61, 735–742, Epub 2012 Mar 28 10.1007/s00011-012-0465-3 22453842

[147] Ezeriņa D., Takano Y., Hanaoka K., Urano Y. and Dick T.P. (2018) N-Acetyl Cysteine Functions as a Fast-Acting Antioxidant by Triggering Intracellular H(2)S and Sulfane Sulfur Production. Cell Chem. Biol. 25, 447.e4–459.e4, Epub 2018 Feb 8 10.1016/j.chembiol.2018.01.011 29429900 PMC6455997

[148] DiNicolantonio J.J., OKeefe J.H. and McCarty M.F. (2017) Boosting endogenous production of vasoprotective hydrogen sulfide via supplementation with taurine and N-acetylcysteine: a novel way to promote cardiovascular health. Open Heart 4, e000600, eCollection 2017. PubMed Central PMCID: PMC547186 10.1136/openhrt-2017-000600 28674632 PMC5471864

[149] Barnado A., Crofford L.J. and Oates J.C. (2016) At the Bedside: Neutrophil extracellular traps (NETs) as targets for biomarkers and therapies in autoimmune diseases. J. Leukoc. Biol. 99, 265–278, Epub 2015 Dec 11. Review 10.1189/jlb.5BT0615-234R 26658004 PMC6608010

[150] Young C.N., Koepke J.I., Terlecky L.J., Borkin M.S., Boyd Savoy L. and Terlecky S.R. (2008) Reactive oxygen species in tumor necrosis factor-alpha-activated primary human keratinocytes: implications for psoriasis and inflammatory skin disease. J. Invest. Dermatol. 128, 2606–2614, Epub 2008 May 8. Erratum in: J Invest Dermatol. 2009 Jul; 129: 1838. Boyd, Savoy L [corrected to Boyd Savoy, L]. PubMed Central PMCID: PMC4102307 10.1038/jid.2008.122 18463678 PMC4102307

[151] Powell C.R., Dillon K.M. and Matson J.B. (2018) A review of hydrogen sulfide (H(2)S) donors: Chemistry and potential therapeutic applications. Biochem. Pharmacol. 149, 110–123, Epub 2017 Nov 23. Review. PubMed Central PMCID: PMC5866188 10.1016/j.bcp.2017.11.014 29175421 PMC5866188

[152] Merighi S., Gessi S., Varani K., Fazzi D. and Borea P.A. (2012) Hydrogen sulfide modulates the release of nitric oxide and VEGF in human keratinocytes. Pharmacol. Res. 66, 428–436, Epub 2012 Jul 27 10.1016/j.phrs.2012.07.002 22842066

